# African signatures of recent positive selection in human *FOXI1*

**DOI:** 10.1186/1471-2148-10-267

**Published:** 2010-09-01

**Authors:** Andrés Moreno-Estrada, Estel Aparicio-Prat, Martin Sikora, Johannes Engelken, Anna Ramírez-Soriano, Francesc Calafell, Elena Bosch

**Affiliations:** 1 Institut de Biologia Evolutiva (UPF-CSIC), Departament de Ciències Experimentals i de la Salut, Universitat Pompeu Fabra, Parc de Recerca Biomèdica de Barcelona, C/Dr. Aiguader 88, 08003 Barcelona, Spain; 2 Centro de Investigación Biomédica en Red de Epidemiología y Salud Pública (CIBERESP), Barcelona, Catalonia, Spain; 3 Department of Genetics, Stanford University School of Medicine, USA

## Abstract

**Background:**

The human *FOXI1 *gene codes for a transcription factor involved in the physiology of the inner ear, testis, and kidney. Using three interspecies comparisons, it has been suggested that this may be a gene under human-specific selection. We sought to confirm this finding by using an extended set of orthologous sequences. Additionally, we explored for signals of natural selection within humans by sequencing the gene in 20 Europeans, 20 East Asians and 20 Yorubas and by analysing SNP variation in a 2 Mb region centered on *FOXI1 *in 39 worldwide human populations from the HGDP-CEPH diversity panel.

**Results:**

The genome sequences recently available from other primate and non-primate species showed that *FOXI1 *divergence patterns are compatible with neutral evolution. Sequence-based neutrality tests were not significant in Europeans, East Asians or Yorubas. However, the Long Range Haplotype (LRH) test, as well as the iHS and XP-Rsb statistics revealed significantly extended tracks of homozygosity around *FOXI1 *in Africa, suggesting a recent episode of positive selection acting on this gene. A functionally relevant SNP, as well as several SNPs either on the putatively selected core haplotypes or with significant iHS or XP-Rsb values, displayed allele frequencies strongly correlated with the absolute geographical latitude of the populations sampled.

**Conclusions:**

We present evidence for recent positive selection in the *FOXI1 *gene region in Africa. Climate might be related to this recent adaptive event in humans. Of the multiple functions of *FOXI1*, its role in kidney-mediated water-electrolyte homeostasis is the most obvious candidate for explaining a climate-related adaptation.

## Background

*FOXI1 *is a family member of the forkhead-box (FOX) transcription factors that are characterized by the FOX ~100 amino acid monomeric DNA-binding domain [1]. Different mutations in the human *FOXI1 *gene and its regulatory binding site on *SLC26A4 *(also known as pendrin) have been shown to compromise the transcription of this anion transporter gene in patients with Pendred Syndrome and nonsyndromic enlargement of the vestibular aqueduct [2]. Foxi1 has also been recognised as a key factor necessary for correct patterning of distal nephron epithelium and adequate acid-base homeostasis in the kidney causing distal renal tubular acidosis in Foxi1^-/- ^mice [3]. Moreover, Foxi1 has been reported to be a crucial activator of the B1-subunit of the vacuolar H+ -ATPase proton pump (*ATP6V1B1*) as well as for pendrin (encoded by *SLC26A4*) and carbonic anhydrase II expression in the epididymal cells, which are required for a correct mouse sperm maturation [4]. Overall, these findings have led to the hypothesis that mutations in the human *FOXI1 *gene might cause sensorineural deafness with distal renal tubular acidosis and male infertility [4].

Thus, *FOXI1 *seems to be involved in the acid-base balance of at least three different organs: inner ear, testis, and kidney. Using human-chimpanzee-mouse orthologous gene trios, *FOXI1 *was suggested as an example of a gene involved in hearing that appeared to present a subset of sites with accelerated amino acid substitution in the human lineage [5]. However, the availability of additional *FOXI *orthologous sequences from a variety of species, including other primates, has permitted this initial finding to be revised. Genes involved in sensory perception show accelerated evolution or are under positive selection in the human lineage [5,6]. Reproduction was only found as a biological function showing an excess of positively selected genes when comparing human and chimpanzee, and thus it could not be established in which of the two branches positive selection acted [7]. Within humans, adaptation to different climates may have entailed adapting kidney function to varying hydration levels. For instance, a thrifty genotype in water retention is suggested to have been selected in the gruelling trans-Atlantic voyage of the slave ships, resulting in a higher prevalence of hypertension in African-Americans [8]. Moreover, variants influencing salt homeostasis in the *CYP3A5 *and *AGT *loci have been shown to be targets of a selective pressure varying in intensity in correlation with latitude [9]. It is of great interest, therefore, to investigate whether *FOX1I *has been the target of local adaptation in humans. In order to test such a hypothesis, we evaluated the patterns of nucleotide variation and looked for signals of positive natural selection in the human *FOXI1 *gene by resequencing 20 Europeans, 20 Asians and 20 Yorubas and by assaying SNP variation in 971 individuals from the Human Genome Diversity Panel (HGDP-CEPH Diversity Panel; [10]).

## Methods

### Population samples

Sixty HapMap DNA samples for resequencing were obtained from Coriell Cell Repositories (Camden, NJ, USA). These consisted of 20 Europeans (Utah residents with ancestry from Northern and Western Europe), 20 Asians (10 Japanese from Tokyo and 10 Chinese from Beijing) and 20 Yorubas (from Ibadan in Nigeria). Coriell Repository numbers for these samples are listed in Table S1 (Additional File 1).

SNP genotypes were obtained for the Human Genome Diversity Panel (HGDP-CEPH), which contains 1,064 DNA samples from individuals representing 51 populations globally distributed [10]. In all statistical analyses atypical, duplicated individuals and deduced first-degree relatives were removed by using the H971 subset recommended by Rosenberg [11]. In order to maximize sample sizes, genotyped samples were re-grouped into 39 populations based on geographic and ethnic criteria as in Gardner et al. [12]. For part of the analysis, populations were further grouped into seven geographical regions: Sub-Saharan Africa (SSAFR), Middle East-North Africa (MENA), Europe (EUR), Central-South Asia (CSASIA), East Asia (EASIA), Oceania (OCE) and America (AME).

All samples used were originally collected with proper informed consent. The research protocol was approved by the Clinical Research Ethical Committee of the Municipal Institute of Health Care (CEIC-IMAS).

### Resequencing of the *FOXI1 *gene

We amplified two overlapping fragments of 2,100 bp and 2,099 bp covering the entire *FOXI1 *genomic sequence. PCRs were performed in a total volume of 25 μl, containing 0.2 mM dNTPs, 1.5 mM MgCl_2_, 0.5 μM of each primer (Additional File 2, Table S2), 1× buffer, 0.05 U Taq polymerase (Ecogen), and 10 ng genomic DNA. PCR conditions were as follows: 3 min at 94°C, 30 cycles of 94°C for 30 sec, 55°C for 30 sec and 72°C for 3 min; and a final step of extension of 5 min at 72°C. PCR products were purified using MultiScreen PCRμ96 chemistry (Millipore) according to the manufacturer's protocol. Amplified PCR products were sequenced using Big Dye Terminator chemistry ver.3.1 (Applied Biosystems) and the sequencing primers listed in Table S2 (Additional File 2). Extension products were purified using the Montage SEQ96 Cleanup kit (Millipore) and run on an ABI 3730 XL sequencer. Sequence analysis and contig assembly for each sample were performed with the Seqman module of the DNASTAR Lasergene software ver. 7.1.0 and visually inspected at least twice. All polymorphic sites were checked manually and heterozygote positions were confirmed by reamplifying and resequencing the SNP site from the same or the opposite strand. Nucleotide sequence data reported are available in the GenBank database under accession numbers GU049150 to GU049269.

### SNP genotyping data

A total of 21 SNPs covering 400 kb centered on the *FOXI1 *gene region were genotyped on the HGDP-CEPH diversity panel. SNPs were selected every 5-10 kb within the gene and up to around 30 kb in both 5' and 3' end flanking regions; from this point, an extra SNP was then added every 40 kb up to around 200 kb in both flanking regions. Preference was given to SNPs with a minor allele frequency (MAF) over 10%, which were compiled from HapMap (Release 7 May 2004) and dbSNP (Build 121 June 2004) databases. SNPs were typed using the SNPlex Genotyping System from Applied Biosystems within a larger set of SNPs covering additional genes as described elsewhere [13]. Illumina HumanHap650K Beadchip genotypes on the HGDP-CEPH panel were downloaded from the Stanford Human Genome Center website. From this publicly available data we extracted a SNP genotype set centered on *FOXI1 *and extending up to 2 Mb (with a total of 693 SNPs), which was complemented by 15 of our previously genotyped SNPs.

### Statistical analysis

In order to test for positive selection in the human lineage we initially applied the improved branch-site test 2 of positive selection [14] on a phylogeny containing five mammalian species (Additional File 3, Figure S1). The human reference sequence for *FOXI1 *and its orthologous sequences in chimpanzee, mouse, rat and dog had been previously extracted from Ensembl (Gene IDs ENSG00000168269, ENSPTRG00000017514, ENSMUSG00000047861, ENSRNOG00000006293, ENSCAFG00000016968) and aligned with ClustalW [15]. The alignment length was 1,137 bp, which became 1,110 bp (97.6%) after removing gaps (Additional File 4, Figure S2). Calculations for the corresponding null and alternative hypotheses were performed using the codeml program implemented in the PAML package [16]. Results for additional likelihood ratio tests of positive selection on the human lineage considering different multispecies alignments were obtained from the Human PAML browser [17]. Table S3 (Additional File 5) summarises the likelihood ratio test results for the branch test (Model H versus Model H null) and for the strict branch positive site test of positive selection (Model A versus Model A null).

Arlequin [18] was used to calculate *F*_*ST *_values among the 39 populations studied with a locus by locus Analysis of Molecular Variance (AMOVA) [19]. Haplotypes were inferred from sequencing data using the Bayesian statistical method in the program PHASE 2.1 [20] using the default parameter set with 1,000 iterations. Haplotype estimation from the 400 kb and 2 Mb SNP genotype data sets was performed with FastPHASE [21]. The Network 4.5.0.1 software package (http://www.fluxus-engineering.com) was used to construct the minimum mutation network by means of the median-joining algorithm [22,23]. The ancestral states for the *FOXI1 *polymorphic positions were inferred from the previously aligned orthologous sequences in chimpanzee, mouse and rat but adding macaque (ENSMMUG00000014124) to the alignment.

Nucleotide diversity statistics and analysis of population polymorphic sites were carried out with the *FOXI1 *resequencing data and the DnaSP software ver. 4.00 [24]. Departures from neutrality were tested by means of Tajima's D, Fu and Li's F, F*, D and D* and Fay and Wu's H tests. In order to obtain realistic distributions for the statistics and thus evaluate evidence for natural selection, we performed 10,000 coalescent simulations using Cosi version 1.1 [25]. As some demographic effects (such as population expansions) and positive selection have similar effects on genealogies [26] those simulations included the ad-hoc, continent-specific human demographic calibration described in Schaffner et al. [25] and that is provided with the Cosi source code. We used an infinite-sites model, with S fixed as the number of segregating sites found, and the length of the simulated sequence was set to the length of the sequence analyzed (4,007 bp). The recombination rates used were those estimated for the region [27]. The critical value for each statistic was obtained from the empirical distribution of the corresponding neutral model with a significance level of 0.05. For the whole human sample, DnaSP software ver. 4.00 [24] was used to produce coalescent neutral simulations with a constant population size.

For every population we analyzed the distribution of the minor allele frequencies (MAF) and the derived allele frequencies (DAF) of the corresponding SNPs along 2 Mb centered on *FOXI1*. The proportion of SNPs with allele frequencies higher or lower than a defined threshold (MAF <0.10 for the MAF analysis and DAF >0.80 for the DAF analysis) was calculated within sliding windows of 100 kb in size every 20 kb and plotted against distance as in Moreno-Estrada et al. [28]. We constructed independent distributions for each population with additional genotype data generated elsewhere [28]. Regions enriched by windows in the top 5% of the corresponding distribution were considered as outliers. Given their small sample size (<10 individuals), the San population was not included in the MAF threshold analysis. Ancestral states for all analyzed SNPs were obtained from the chimpanzee and/or the macaque genome sequences (panTro2, Mar. 2006 assembly and rheMac2, Jan. 2006 assembly, respectively).

Unusually long range haplotypes along the 2 Mb region centered on *FOXI1 *were explored by applying two complementary EHH-based approaches especially designed to detect intermediate frequency and fixed (or nearly so) selected variants. For the first approach, we applied both the Long Range Haplotype (LRH) test [29] and the iHS statistic [30], and for the second we used the XP-Rsb statistic [28]. The LRH test was carried out using the SWEEP software package (version 1.1) defining cores as the longest non-overlapping core haplotypes with at least one SNP and not more than 20 SNPs. In order to obtain additional data for a background distribution of EHH values we used phased haplotype data for eleven 2 Mb regions studied elsewhere [28]. For each core haplotype identified, we calculated the EHH and the relative EHH (REHH) at varying distances from the core for each main continental region separately. More specifically, marker H (defined as the degree to which each added marker at a further distance causes the extended haplotype to decay for all core haplotypes) of 0.04 and 0.02 were selected as they are roughly equivalent to a genetic distance of 0.25 and 0.3 cM, respectively (matching the observed amount of recombination in the actual tested data). Core haplotypes were placed in 5% frequency bins and the respective EHH and REHH values were log-transformed for each bin in order to obtain approximately normally distributed values. The empirical significance of the LRH test was estimated by means of p and q values as in Moreno-Estrada et al. [28]. The Integrated Haplotype Score (iHS) was estimated as previously described [30,31] using publicly available genotype data for ~650,000 genome-wide distributed SNPs in 39 populations from the HGDP-CEPH diversity panel [32]. With an approach similar to that described by Nielsen et al. [33] to detect regions with aberrant allele frequency spectra (test 1), we applied a composite likelihood test to detect regions with aberrant "iHS spectra". We first divided |iHS| in bins of size 0.1, and then estimated the probability that a SNP had an |iHS| value in a particular bin, both for each SNP in the whole genome dataset (which produced an empirical background distribution) as well as for each sliding window of 31 SNPs over the whole genome. Two composite likelihoods were estimated for each window, one by multiplying the probabilities obtained from the window itself, and the other by multiplying the probabilities obtained from the whole genome. A log-likelihood ratio was then formed comparing both likelihoods, where extreme values indicate unusual distributions of iHS in the respective window compared to the rest of the genome. For each SNP this log-likelihood ratio for the window centered on the SNP is referred to as windowed iHS in Results. Finally, in order to allow for multiple population comparisons of EHH, we computed XP-Rsb for every SNP site and population *versus *all other HGDP-CEPH populations as described in Moreno-Estrada et al. [28]. In order to assess significance, we obtained a p-value for each population and SNP site along the *FOXI1 *region by ranking its XP-Rsb value with respect to the genome-wide distribution generated with the ~650,000 SNPs from Li et al. [32]. We then log transformed the p-values and plotted them against position, searching for clusters of significant values inside or around the *FOXI1 *gene.

## Results

### *FOXI1 *divergence patterns

The recent increase of available newly sequenced genomes led us to re-evaluate the initial evidence of non-neutral evolution of *FOXI1 *in the human lineage obtained with three-species sequence alignments [5]. When considering a phylogeny of five mammals, the improved branch-site test 2 of positive selection [14] rejected the hypothesis of positive selection at a subset of sites in the human branch (p = 0.7039). We also investigated the patterns of FOXI1 protein evolution from several multiple sequence alignments, including additional non-human primates besides chimpanzee. We used two maximum likelihood methods (see Materials and Methods) to specifically test for: (i) a d_N_/d_S _ratio on the human branch significantly different from 1, and (ii) codon sites with a d_N_/d_S _ratio significantly different from 1 in the human lineage. Overall *FOXI1 *did not present significantly accelerated amino acid substitution rates or particular codon sites undergoing positive selection in the human branch in any of the comparisons (Additional file 5, Table S3).

### Patterns of *FOXI1 *sequence variation

Polymorphic variation in the *FOXI1 *gene was investigated by sequencing 4,007 bp, encompassing its two exons, most of its untranslated regions, and its corresponding intronic region, in 20 Yorubas, 20 Asians and 20 Europeans. We found 22 sequence variations: 21 substitutions and one deletion/insertion polymorphism (Table 1). Among them, six were singletons: one in Yorubas, three in Europeans and two in Asians. One of the Asian-specific singletons was the only non-synonymous substitution observed (rs3828625), an Asn to Ser replacement at amino acid position 362 for isoform a (or amino acid position 267 for isoform b). The remaining three exonic polymorphisms were synonymous changes. Twenty of the 22 sequence variations detected here had been previously reported in dbSNP build 131. Excluding singleton variants, we identified a total of 15 haplotypes (Table 2). Half of the analysed chromosomes belonged to either Ht-01 or Ht-04, found in all three populations studied. We found three specific haplotypes for Africans, three for Europeans, but none specific for Asians. In order to visualize the phylogenetic relationships among the identified haplotypes we constructed a median-joining network; ht-08 carried ancestral alleles at each SNP (Additional File 6, Figure S3). The reticulated pattern observed in the network points to the action of recurrent mutation or recombination. However, given that more than one nucleotide position is often involved, the latter seems more likely as a mechanism for producing new sequence variation.

**Table 1:** Summary of *FOXI1 *polymorphisms

					Derived Allele Frequency			
N°	**Position**^**a**^	dbSNP code	**Type of change**^**b**^	**Nucleotide change**^**c**^	Yoruban (2N = 40)	European (2N = 40)	Asian (2N = 40)	**F**_**ST**_^**d**^
1	169465818	rs2277944	CS	G/A	0.58	0.20	0.35	0.123
2	169466184	rs2277945	I	T/C	0.23	0.28	0.43	0.026
3	169466367	rs2112669	I	C/G	0.55	0.23	0.33	0.092
4	169466686	rs11951903	I	T/C	0.05	0.00	0.25	0.162
5	169466751	rs4315934	I	A/G	0.88	0.55	0.78	0.113
6	169466837	rs4315935	I	A/G	0.88	0.55	0.78	0.113
7	169467111	rs2879269	I	A/G	0.03	0.10	0.00	0.042
8	169467123	rs7380481	I	T/C	0.23	0.28	0.43	0.026
9	169467572	rs77136537	I	G/A	0.00	0.03	0.00	0.000
10	169468070	rs55685928	CS	G/A	0.05	0.00	0.00	0.026
11	169468100	rs10063424	CS	C/T	0.10	0.10	0.00	0.028
12	169468141	rs3828625	CNS	A/G	0.00	0.00	0.03	0.000
13	169468312	rs6873124	UTR	C/A	0.53	0.20	0.33	0.091
14	169468336	rs55762796	UTR	A/T	0.05	0.08	0.03	-0.012
15	169468524	rs72828668	UTR	G/C	0.00	0.00	0.03	0.000
16	169468633	rs6555887	UTR	A/G	0.15	0.10	0.00	0.051
17	169468728	rs6555888	UTR	A/G	0.83	0.70	0.95	0.082
18	169468749	rs77823283	UTR	A/G	0.00	0.03	0.00	0.000
19	169468769		UTR	T/A	0.00	0.03	0.00	0.000
20	169469034		UTR	T/A	0.03	0.00	0.00	0.000
21	169469117	rs45466695	UTR	C/T	0.000	0.08	0.00	0.051
22	169469123	rs3839285	UTR	-/T	0.53	0.20	0.33	0.091

^a^Positions are based on NCBI build 36.3

^b^CNS stands for coding non-synonymous; CS, synonymous; I, intronic; and UTR, untranslated region

^c^The allele named first corresponds to the ancestral state for each SNP

^d^Overall F_ST _among Yorubans, Europeans and Asians

**Table 2:** Summary of human *FOXI1 *haplotypes

	1	2	3	4	5	6	7	8	10	11	13	14	16	17	21	22				
Anc	G	T	C	T	A	A	A	T	G	C	C	A	A	A	C	-	Europe	Africa	Asia	Total
Ht-01	A	.	G	.	G	G	.	.	.	.	A	.	.	G	.	T	8	19	3	30
Ht-02	A	.	G	C	G	G	.	.	.	.	A	.	.	G	.	T	0	2	10	12
Ht-03	A	C	.	.	G	G	.	C	.	.	.	.	.	G	.	.	0	1	1	2
Ht-04	.	C	.	.	G	G	.	C	.	.	.	.	.	G	.	.	10	8	16	34
Ht-05	.	C	.	.	G	G	.	C	.	.	.	.	.	.	.	.	1	0	0	1
Ht-06	.	.	.	.	G	G	.	.	.	.	.	T	.	G	.	.	3	2	1	6
Ht-07	.	.	.	.	.	.	.	.	.	.	.	.	.	G	.	.	7	1	7	15
Ht-08	.	.	.	.	.	.	.	.	.	.	.	.	.	.	.	.	6	1	2	9
Ht-09	.	.	G	.	.	.	.	.	.	.	.	.	.	.	.	.	1	0	0	1
Ht-10	.	.	.	.	G	G	.	.	.	.	.	.	G	.	.	.	0	2	0	2
Ht-11	.	.	.	.	.	.	.	.	.	T	.	.	G	.	.	.	0	1	0	1
Ht-12	.	.	.	.	.	.	.	.	A	T	.	.	G	.	.	.	0	1	0	1
Ht-13	.	.	.	.	.	.	G	.	.	T	.	.	G	.	.	.	1	1	0	2
Ht-14	.	.	.	.	.	.	G	.	.	T	.	.	G	.	T	.	3	0	0	3
Ht-15	A	.	G	.	G	G	.	.	A	T	.	.	G	.	.	.	0	1	0	1

Note: Each polymorphic variant is displayed below the corresponding ancestral position. Ancestral-like alleles are indicated with dots.

Summary statistics of genetic diversity and neutrality tests for *FOXI1 *are reported in Table 3. These levels of diversity are in agreement with estimates from other genomic regions sequenced in a similar set of samples (SeattleSNPs database, http://pga.gs.washington.edu/). Notably, in the Yoruban population the number of segregating sites, and different haplotypes as well as values of nucleotide diversity were similar to the European population sample. However, in Africans haplotype diversity was lower. In order to investigate the possible genetic footprint of selection we performed several neutrality tests on each of these three populations and on the whole human sample (Table 3). Significance was estimated by means of coalescent simulations under each inferred past demography [25] or in the case of the whole human sample by considering constant population size. None of the neutrality statistics displayed significant departure from neutrality (P > 0.05).

**Table 3:** Population sequence variation and neutrality test statistics for *FOXI1*

Population	**2N**^**a**^	**S**^**b**^	**π**^**c**^	**K**^**d**^	**H**^**e**^	Tajima's D	Fu and Li's D*	Fu and Li's D	Fu and Li's F*	Fu and Li's F	Fu's Fs	Fay and Wu's H
European	40	17	0.0012 ± 0.0001	12	0.874 ± 0.026	0.485	0.431	0.433	0.530	0.543	-0.421	1.600
Asian	40	13	0.0010 ± 0.0001	8	0.768 ± 0.045	1.134	0.065	0.030	0.486	0.485	1.969	-1.923
Yoruban	40	16	0.0011 ± 0.0001	12	0.741 ± 0.063	0.487	0.763	0.799	0.793	0.831	-0.664	-2.928
All	120	22	0.0012 ± 0.0001	19	0.837 ± 0.019	0.360	-0.808	-0.863	-0.430	-0.458	-1.417	-0.410

Note: At P = 0.05, none of the neutrality statistics were significant

^a^Number of chromosomes

^b^Number of segregating sites

^c^Nucleotide diversity per base pairs

^d^Total number of haplotypes

^e^Haplotype diversity

### Patterns of SNP variation in *FOXI1*

**Figure 1:**
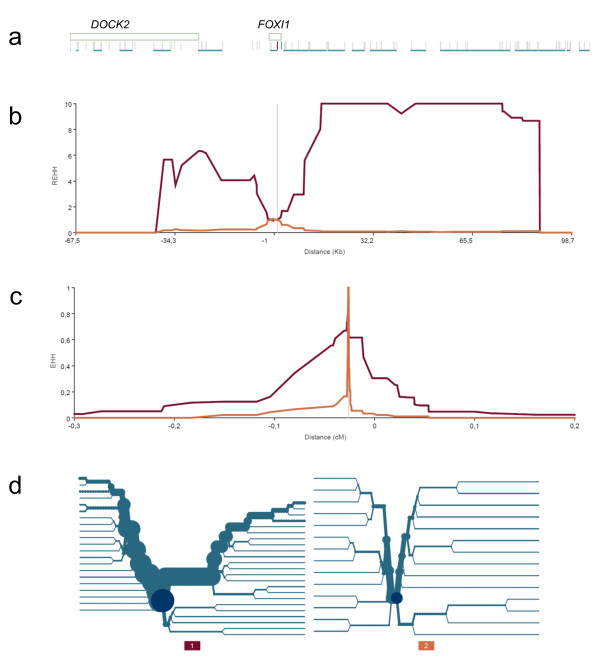
**Decay of linkage disequilibrium around *FOXI1 *in Africans**. (A). Genes and SNPs in the region. Boxes represent genes and vertical gray lines are SNPs. Underlined SNPs are those constituting core haplotypes. (B). Relative EHH over physical distance. The vertical grey line indicates the core region from which REHH is measured. Red and orange lines show REHH values at increasing distances from the core for the two main extended haplotypes. (C). Breakdown of EHH over genetic distance. The vertical grey line indicates the core region from which EHH is measured. Red and orange lines show EHH values at increasing distances from the core for the two main extended haplotypes (D) Haplotype bifurcation plots for each core haplotype. The dark blue circle in the center of each plot indicates the core and each node represents a SNP (and thus, a possible bifurcation). Blue lines indicate the observed haplotypes and the thickness of the branch is proportional to the haplotype frequency in the population.

**Figure 2:**
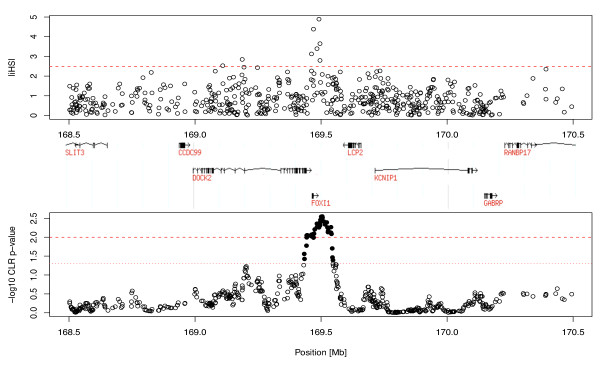
**iHS values against physical distance in the Sub-Saharan African continental sample**. iHS values for individual SNPs flanking the *FOXI1 *region (2 Mb) against physical distance (top panel) and p-values for the composite likelihood ratio (CLR) test based on a 31-SNP sliding window analysis to detect local regions enriched for high iHS values (bottom panel). Solid circles above the dotted line indicate P-values below 0.05 genome-wide significance level, and the dashed line indicates the P = 0.01 threshold.

**Figure 3:**
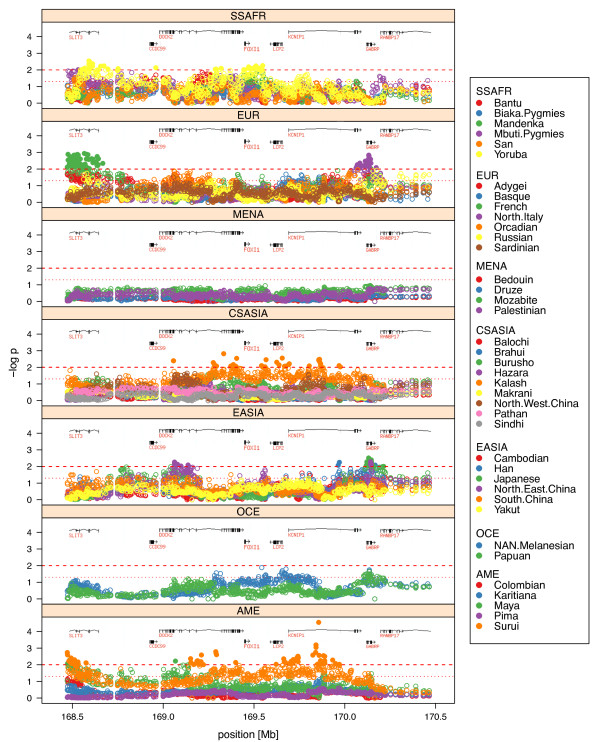
**Distribution of -log p-values for XP-Rsb across 39 HGDP-CEPH populations grouped into major geographical regions**. The genome-wide significance of the XP-Rsb statistic per SNP site and population is plotted against distance over 2 Mb flanking the *FOXI1 *gene. Location of genes is shown on top of each plot. Dotted and dashed lines show 0.05 and 0.01 significance levels, respectively. Values below 0.01 significance level are additionally represented with solid color circles. Populations are shown according to their continental groups: Sub-Saharan Africa (SSAFR), Europe (EUR), Middle East-North Africa (MENA), Central-South Asia (CSASIA), East Asia (EASIA), Oceania (OCE) and America (AME).

In order to widen both the genomic context and the range of human populations analyzed, we also explored the pattern of SNP variation along a 2 Mb region centered on *FOXI1 *in 39 worldwide human populations covering more human genetic diversity than the three samples we sequenced. In particular, we looked for an excess of low frequency variants, the presence of high frequency derived alleles, and unusually long range haplotypes. These possible signatures of natural selection persist in the human genome at varying time scales, and therefore give information about adaptative events occurring at different intervals during our evolutionary history [34]. For each population and within multiple 100 kb sliding windows along the *FOXI1-*centered 2 Mb region we separately plotted: (i) the proportion of SNPs with MAF <0.10 (Additional File 7, Figure S4) and (ii) the proportion of SNPs with DAF >0.80 (Additional File 8, Figure S5). In both analyses, no particular pattern emerged around or surrounding the *FOXI1 *gene in any of the populations studied. However, when searching for signatures of unusually long haplotypes, we found strong signals from all the three statistics we applied. First, according to the LRH test [29], up to five different core haplotypes close to *FOXI1 *clearly stood out with relatively high frequencies (>0.50) and significant REHH values in the Yoruba population (see Table 4 and Materials and Methods). A picture of the unusual EHH breakdown around *FOXI1 *in Yorubas is presented in Figure 1. Similarly, in Sub-Saharan Africans (SSAFR) from the HGDP-CEPH diversity panel, the complete *FOXI1 *gene region was clearly enriched for outliers of the iHS statistic [30], reaching significant values within the top 1% of the genome-wide distribution (iHS >3) (see Figure 2). The strongest signals were located inside *FOXI1 *and extended several kb downstream in agreement with the location of the most significant core haplotypes observed with the LRH test described above. When ranking all genes in the genome by their maximum windowed iHS within the gene region (defined as the longest transcript ± 2 kb), *FOXI1 *was ranked number 339 out of 17,637 (p-value = 0.019) in the SSAFR continental sample. Finally, we used the XP-Rsb statistic [28] to compare the integrated EHH at each particular SNP site in a population with that averaged across multiple populations (as opposed to comparing the EHH of alleles within the same population like in the LRH test and the iHS statistic). Interestingly, a cluster of significant XP-Rsb values was detected in the *FOXI1 *region for the Yoruba (p < 0.01) and Mandenka (p < 0.05) African populations. Figure 3 shows genome-wide significance for XP-Rsb at every SNP site and population plotted against distance along the 2 Mb region centered in *FOXI1*. The cluster of significant values involving *FOXI1 *was exclusively observed in Africans and also correlates with the location of the LRH and iHS signals previously described. Other significant values can be seen in Figure 3, for example, at 168.5 Mb for the French population, and around 170 Mb for N. Italy. These signals lie well beyond *FOXI1 *and, given the pattern of linkage disequilibrium in the region, are most certainly unrelated to this gene.

**Table 4:** Core haplotypes with significant REHH values in Africans involving *FOXI1*

**H**^**a**^	REHH	Frequency	**Distance (bp)**^**b**^	**Distance (cM)**^**c**^	Core haplotype	**Genes in core region**^**i**^	*P *value	***q *value**^**j**^
0.02	19.58	0.528	80443	0.056	ACCC^d^	*DOCK2, FOXI1*	0.8 × 10^-5^	0.0038
0.04	16.93	0.528	61086	0.026	ACCC^d^	*DOCK2, FOXI1*	0.3 × 10^-5^	0.0016
0.04	12.08	0.590	-64129	-0.058	AGC^e^	*FOXI1*	0.6 × 10^-4^	0.0213
0.04	8.32	0.675	-41241	-0.051	AG^f^	*DOCK2, FOXI1*	0.8 × 10^-4^	0.0247
0.04	10.42	0.528	55531	0.028	A^g^	*DOCK2, FOXI1*	0.8 × 10^-4^	0.0191
0.04	10.65	0.561	-54967	-0.053	CTG^h^	*DOCK2, FOXI1*	1.2 × 10^-4^	0.0215

^a^Degree to which each added marker at a further distance causes the extended haplotypes to decay for all core haplotypes

^b^Physical distance (bp) from the core at which the signal has been captured. (-) indicates downstream direction. Otherwise upstream

^c^Genetic distance (cM) from the core at which the signal has been captured. (-) indicates downstream direction. Otherwise upstream

^d^rs7736379, rs6872596, rs4449553, rs17562083

^e^rs7729440, rs2879278, rs11134616

^f^rs4867919, rs11134612

^g^rs1501644

^h^rs7709558, rs12515896, rs6861611

^i^Genes within ±100 kb around the core are considered

^j^Expected proportion of false positives among all significant p values

We next explored what may have been the functional variation underlying the recent selection signal detected in Africa by these two EHH-based approaches. To that effect, using HapMap data [35] we studied the linkage disequilibrium (LD) pattern around *FOXI1 *in the Yoruban population and found a LD block containing *FOXI1 *in a region of ~70 kb between two hotspots of recombination. As shown in Figure S6 (Additional File 9), a similar LD block structure was detected in the Yoruban population of the HGDP-CEPH panel. Then we screened all known SNP variation for a putative functional effect using PupaSuite [36,37] in a ~140 kb segment containing the previously identified LD block and all the significant core haplotypes found in the *FOXI1 *gene region (see Additional File 9, Figure S6). Out of 646 SNPs we detected 19 as functionally relevant polymorphisms (Additional File 10, Table S4). Since we have found that the footprint of natural selection seems to be evident in Africans, we can narrow down that candidate list by rejecting the SNPs with small frequency differences between Africans and non-Africans. After removing the SNPs with an absolute difference in MAF between Africans and Europeans smaller than 0.2, only six SNPs remained. Five of them affect exonic splicing enhancers either for the *DOCK2 *gene (rs6555882, rs1045176 and rs9307) or the *FOXI1 *gene (rs2277944 and rs6873124), while rs7704953 creates a new transcription factor binding site for *FOXI1. *Except for rs6555882, which maps further away, these SNPs are all within the 70 kb block described above. Therefore, they (or unknown functional variants in linkage disequilibrium with them) are the best candidates to represent the functional variation that caused this signature of selection.

**Figure 4:**
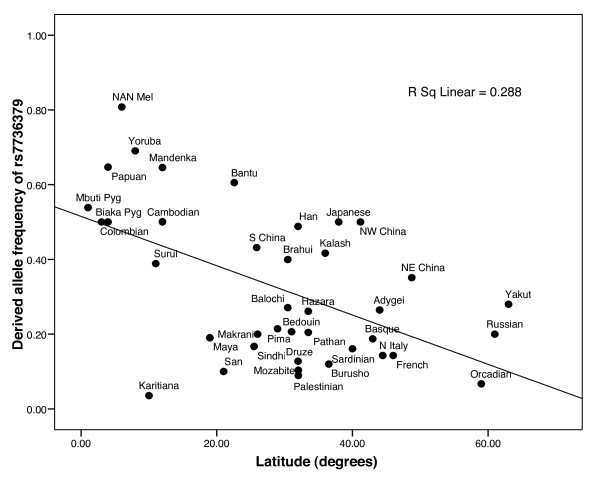
**Allele frequencies at rs7736379 as a function of absolute latitude in 39 HGDP-CEPH populations**. The line represents linear regression.

Finally, in order to explore a possible relation between climate and *FOXI1 *variation, we tested for correlation between allele frequencies and absolute latitude for the SNPs in the significant core haplotypes in the LRH test, SNPs with significant values for the iHS and XP-Rsb statistics, and rs2277944 (worldwide allele frequency distributions were not available for the other suggested candidate functional SNPs). Out of 27 SNPs, correlation with absolute latitude was nominally significant (p < 0.05) for 13 of them. All significant core haplotypes contained at least one SNP with significant correlation with absolute latitude (p < 0.05), and rs2277944 was also significantly correlated with latitude (p < 0.01). The most significant correlation (Pearson's r = -0.536, p < 0.01 after Bonferroni correction) was for allele A at rs7736379 in core ACCC (see Table 4), implying that its frequency decreases towards higher latitudes (Figure 4). Since the most significant event to shape human genetic diversity was the Out of Africa migration, we recalculated partial correlation coefficients with absolute latitude by controlling for the geographical distance to East Africa (Nairobi). All the significant correlations mentioned above remained significant, implying that the dispersion out of Africa is not a meaningful confounding factor for this result. However, when put in a genomic context, these correlations are not exceptional: out of 585,379 SNPs with minor allele frequency >0.05 genotyped in the same individuals by Li et al. [32], 8.6% had more extreme correlations with absolute latitude, and 14% after correcting for distance to East Africa.

## Discussion

Genes related to fertility and/or sensory perception may have been driven by accelerated evolution in humans, but, contrary to Clark et al. [5], we did not find in the human branch such acceleration of the *FOXI1 *gene, which is involved in the function of the ear, testis, and kidney [8]. It is recognised that the accuracy and power of likelihood ratio tests (LRTs) for detecting positive selection improve with the number of species used in such approaches [38]. Consequently, we used the Human PAML browser [17] in order to perform both branch and branch-site tests of positive selection in multiple species comparisons containing several mammals and additional primates to human and chimpanzee. None of the tests performed indicated positive selection for the *FOXI1 *coding sequence in the lineage leading to humans. However, ω is ~1 in many branches of the *FOXI1 *phylogeny (see Additional File 3, Figure S1), which suggests that the *FOXI1 *amino acid replacement rate is equally fast in all branches and that comparative LRTs such as the ones performed here may lack power to detect positive selection in any particular lineage. In any case, we did not find evidence of a specific evolution pattern for *FOXI1 *in the lineage leading to humans.

Variation in the *FOXI1 *gene sequence was compatible with neutral evolution in samples of Africans, East Asians and Europeans. However, patterns of extended linkage disequilibrium in the *FOXI1 *gene region suggested recent positive selection in some of the African samples of the HGDP-CEPH diversity panel [10]. Half of the SNPs with significant values for the iHS or XP-Rsb statistics and of those contained in significant cores haplotypes in the LRH test correlated with latitude, suggesting a possible role for climate in the adaptation mediated by *FOXI1*. A higher correlation with latitude may not have been reached due to the apparent recentness of the selective event and its restriction to Sub-Saharan African populations. An alternative explanation (but less likely, given the distance to most of the significant signals of selection found) is geographically localized selection at the *DOCK2 *gene, which encodes for a cytoplasmatic protein required for lymphocyte chemotaxis in response to chemokines [39]. It is suggested that patterns of linkage disequilibrium pinpoint recent selective events, while the accumulation of variation by mutation requires some time before sequence-based neutrality statistics become significant [40]. The finding of no significant deviation from neutral evolution with statistics such as Tajima's D, or Fay and Wu's H has been previously recognized to be consistent with the low power of these traditional tests to detect recent selective sweeps as for example in the case of the *G6PD *locus [29]. Moreover, tests based on the allele frequency spectrum have low power to detect incomplete sweeps, especially after a selective event in standing genetic variation [40]. Interestingly, while nucleotide and haplotype diversities tend to be higher in Africans than in other populations, this was not the case for *FOXI1*, which could be taken as further evidence of positive selection in Africans. No non-synonymous coding SNPs were found in the core haplotypes or their linked variation. Therefore, taken as a whole, our results suggest that recent selection may have acted on the expression rather than on the amino acid sequence of *FOXI1*. Based on two extensive collections of microarray data from a large variety of available human tissue samples, Genevestigator [41] and GeneSapiens [42], *FOXI1 *has the highest mRNA expression levels in kidney (but inner ear data was not available). Fittingly, among the diverse functions of this transcription factor, its role in the kidney as a master regulator of vacuolar H+-ATPase proton pump subunits [8] might be an intriguing candidate function for climate adaptation through water-electrolyte homeostasis and prevention of dehydration. If that were the case, *FOXI1 *would join the ranks of the growing number of known genes such as *FABP2*, *RAPTOR *and *SLC24A5 *[43] that allowed humans to adapt to the diversity of climates we encountered during our past expansion.

## Conclusions

We have found no genetic evidence of accelerated protein evolution for the *FOXI1 *gene in the human lineage; however, within humans we have discovered signatures of a recent episode of positive selection in African populations. Our evolutionary approach identifies directions for future functional analyses of how genetic variation in the *FOXI1 *gene region can result in phenotypic differences among human populations, which might be related to water-electrolyte homeostasis.

## Authors' contributions

AME participated in the design of the study, carried out the analysis of the SNP and resequencing data and helped to draft the manuscript. EAP performed the resequencing analysis. MS, JE and ARS participated in the statistical analysis. FC helped in the statistical analysis and in drafting the manuscript. EB participated in the design, analysis and coordination of the study and drafted the manuscript. All authors read and approved the final manuscript.

## Supplementary Material

Additional file 1 **Table S1: Coriell repository numbers**. Click here for file

Additional file 2 **Table S2: Amplification and sequencing primers**. Click here for file

Additional file 3 **Figure S1: Phylogeny of the five mammalian species used in PAML analysis**. Each branch is labeled with the corresponding estimate of ω calculated under a free branch model [44] with the codeml program within PAML using standard parameters [28]. Inferred synonymous and non-synonymous substitutions are indicated within brackets. Click here for file

Additional file 4 **Figure S2: Alignment of the five *FOXI1 *mammalian sequences used in PAML analysis**. Click here for file

Additional file 5 **Table S3: Significance of the likelihood ratio tests of positive selection performed on the human lineage for the *FOXI1 *gene**. Click here for file

Additional file 6 **Figure S3: Median Joining Network of human *FOXI1 *haplotypes**. Nodes in the median joining network are proportional to frequencies and branch lengths to the number of polymorphic base substitutions. Click here for file

Additional file 7 **Figure S4: Distribution of low-frequency minor alleles**. The proportion of SNPs with a minor allele frequency (MAF) of less than 0.10 within 100 kb sliding windows is plotted for each population. Solid dots represent values above the 95th percentile for each population, whereas open dots are values below the 95th percentile. Click here for file

Additional file 8 **Figure S5: Distribution of high-frequency derived alleles**. The proportion of SNPs with a derived allele frequency (DAF) greater than 0.80 within 100 kb sliding windows is plotted for each population. Solid dots represent values above the 95th percentile for each population, whereas open dots are values below the 95th percentile. Click here for file

Additional file 9 **Figure S6: Pattern of linkage disequilibrium around *FOXI1 *in the Yoruba population from the CEPH-HGDP diversity panel**. Linkage disequilibrium from position 169,401,507 to 169,544,856 on chromosome 5, NCBI build 36.3. Green boxes represent significant core haplotypes from the LRH test and are labelled with letters d-h as in Table 4. Click here for file

Additional file 10 **Table S4: Functional characterization and allele frequencies for functionally relevant SNPs within ~ 140 kb containing the *FOXI1 *gene**. Click here for file
