# Supplementary material for: African signatures of recent positive selection in human FOXI1
Source: BMC Evol Biol. 2010 Sep 1;10:267. doi: 10.1186/1471-2148-10-267 (PMC2939579; doi:10.1186/1471-2148-10-267)
Supplement: Additional file 1 — Table S1: Coriell repository numbers. [file 1471-2148-10-267-S1.PDF]

Table S1. Coriell repository numbers.

Population	Coriell repository numbers
Yoruban	NA18501, NA18502, NA18507, NA18508, NA18855, NA18856, NA18861, NA18862, NA19127, NA19128, NA19137, NA19138, NA19171, NA19172, NA19203, NA19204, NA19206, NA19207, NA19209, NA19210
European	NA06994, NA07000, NA07345, NA07357, NA11829, NA11830, NA11839, NA11840, NA11992, NA11993, NA12003, NA12004, NA12043, NA12044, NA12056, NA12057, NA12750, NA12751, NA12812, NA12813
Han Chinese	NA18576, NA18577, NA18579, NA18582, NA18593, NA18623, NA18624, NA18632, NA18635, NA18636
Japanese	NA18940, NA18942, NA18943, NA18944, NA18948, NA18949, NA18951, NA18956, NA18970, NA18973
