# Supplementary material for: African signatures of recent positive selection in human FOXI1
Source: BMC Evol Biol. 2010 Sep 1;10:267. doi: 10.1186/1471-2148-10-267 (PMC2939579; doi:10.1186/1471-2148-10-267)
Supplement: Additional file 2 — Table S2: Amplification and sequencing primers. [file 1471-2148-10-267-S2.PDF]

Table S2. Amplification and sequencing primers.

Primer ID	Sequence (5´ - 3´)
Amplification	
FO-F1	GTCATTAGTGGGGACCTGAG
FO-R1	GGCATGAGCATTAAGGAGTT
FO-F2	GCCAGGACTCAAGTCTGTCT
FO-R2	GCACCACATGTTTGTTTGTT
Sequencing	
FO-S1F	CAACCCCTACCTCTGGTTC
FO-S2R	AGAGCCGAGTAGGAATAGGG
FO-S3F	GTCCCCAAGGAAGACTCAC
FO-S4F	TTGGTGAATGAATGACTGGA
FO-S5F	CTCAAAGGAACCCCAACTC
FO-S6F	GAGTCCCAGAGTTTTCCTGA
FO-S7F	TTTACCCCCTTTCACTTTTG
FO-S8F	TGACCTTCAACTCCTTCTCC
FO-S9F	CAGAGCAGCACTAACAGTGG
FO-S10F	GCTACCACTCAAGGAAGGAA
