# Supplementary figures and images for: African signatures of recent positive selection in human FOXI1
Source: BMC Evol Biol. 2010 Sep 1;10:267. doi: 10.1186/1471-2148-10-267 (PMC2939579; doi:10.1186/1471-2148-10-267)

**Figure S1. Phylogeny of the five mammalian species used in PAML analysis.**

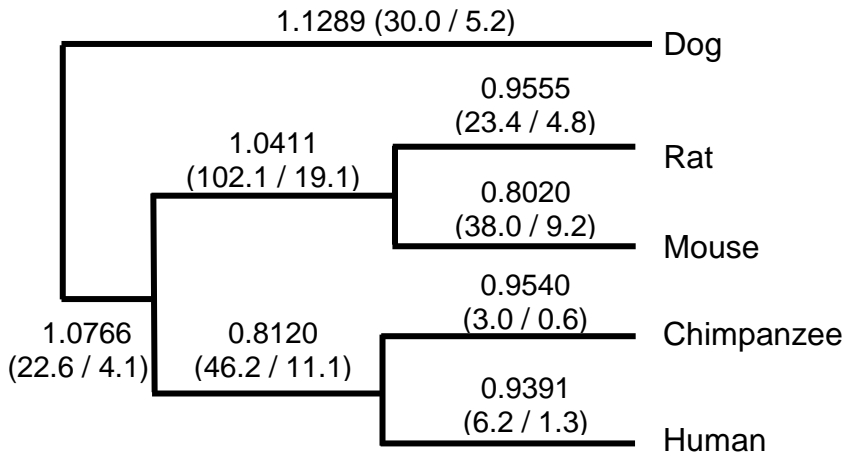

Supplement: Additional file 3 — Figure S1: Phylogeny of the five mammalian species used in PAML analysis. Each branch is labeled with the corresponding estimate of ω calculated under a free branch model [44] with the codeml program within PAML using standard parameters [28]. Inferred synonymous and non-synonymous substitutions are indicated within brackets. [file 1471-2148-10-267-S3.PDF]

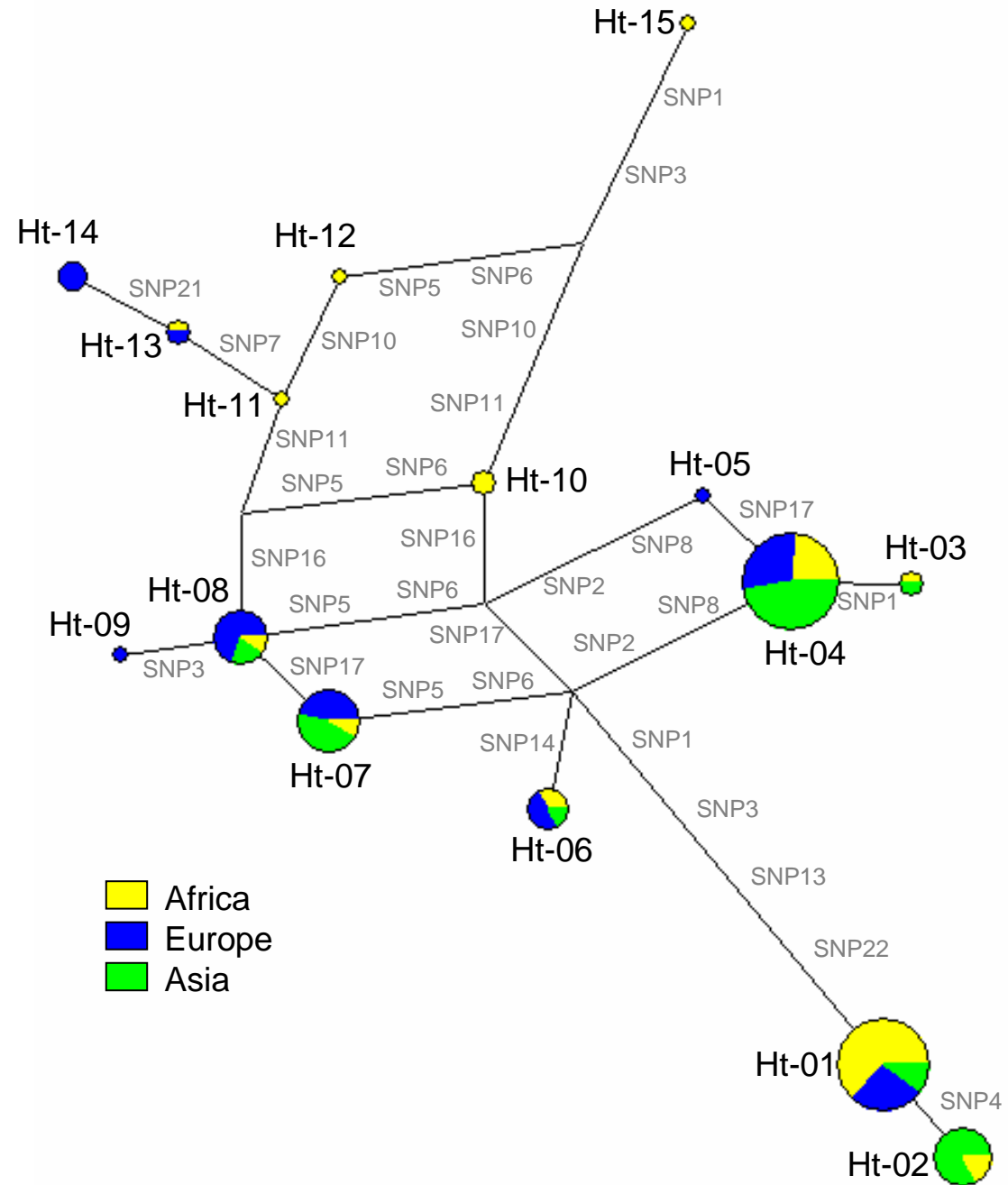

Supplement: Additional file 6 — Figure S3: Median Joining Network of human FOXI1 haplotypes. Nodes in the median joining network are proportional to frequencies and branch lengths to the number of polymorphic base substitutions. [file 1471-2148-10-267-S6.PDF]

MAF &lt; 0.1

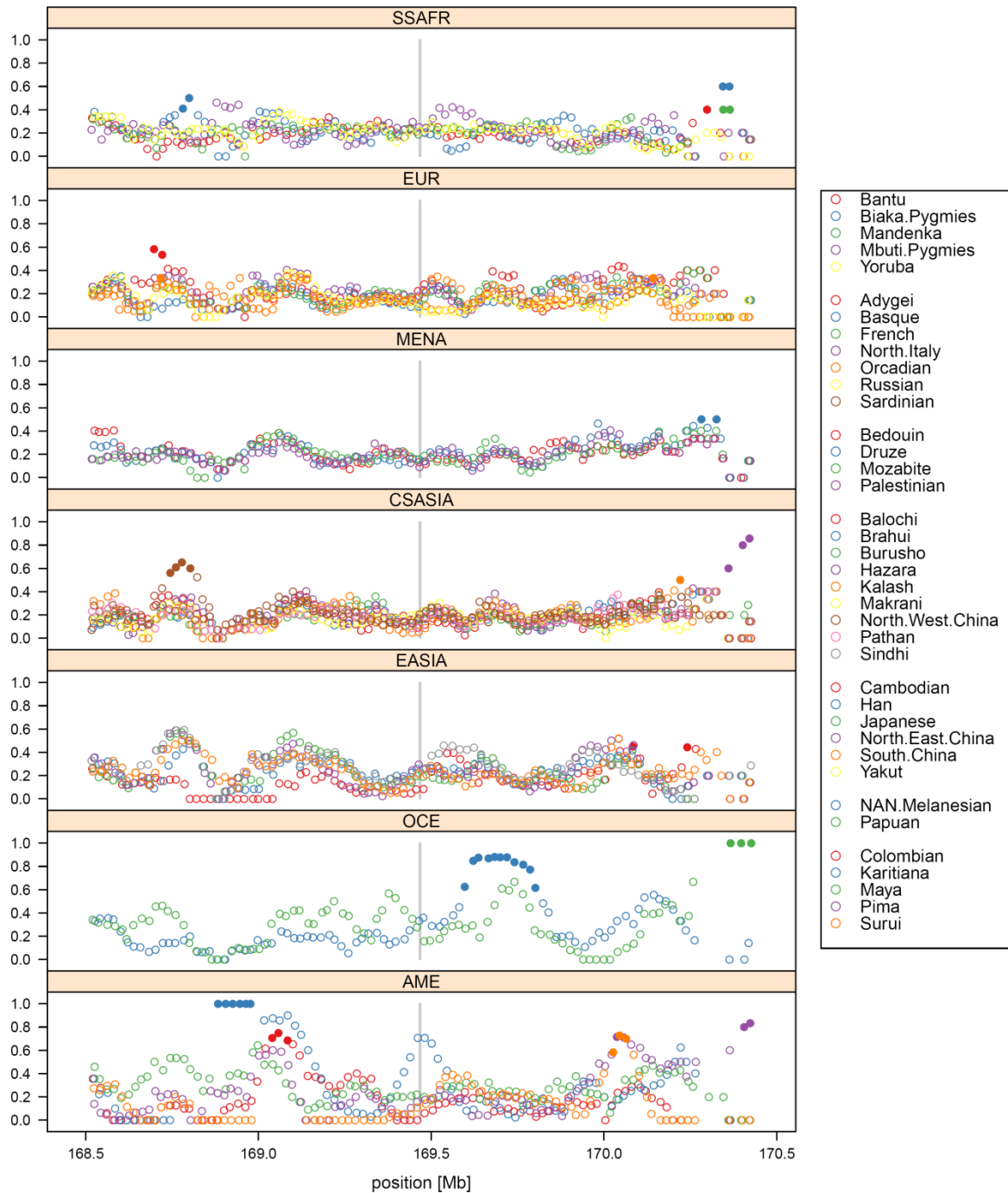

Supplement: Additional file 7 — Figure S4: Distribution of low-frequency minor alleles. The proportion of SNPs with a minor allele frequency (MAF) of less than 0.10 within 100 kb sliding windows is plotted for each population. Solid dots represent values above the 95th percentile for each population, whereas open dots are values below the 95th percentile. [file 1471-2148-10-267-S7.PDF]

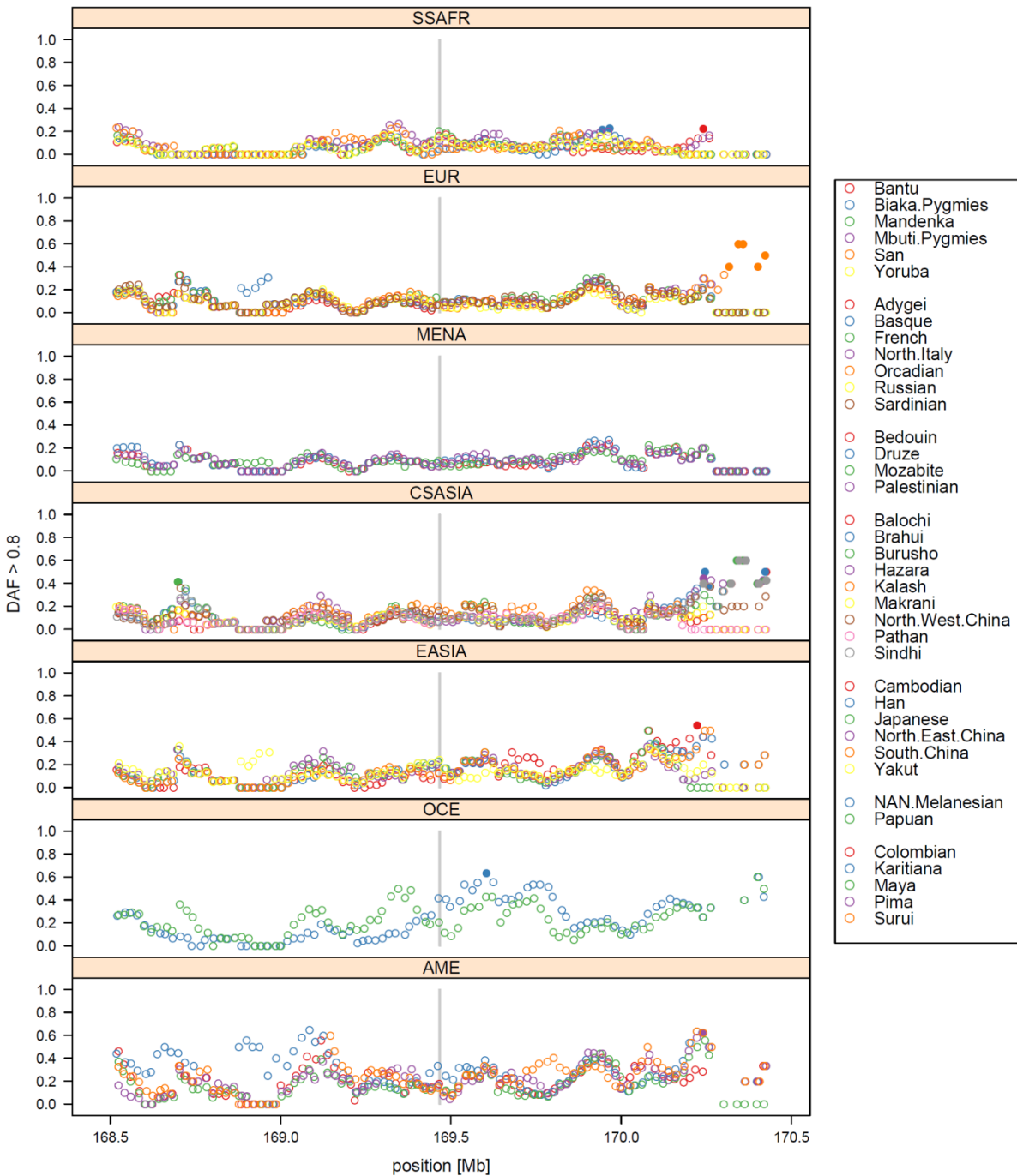

Supplement: Additional file 8 — Figure S5: Distribution of high-frequency derived alleles. The proportion of SNPs with a derived allele frequency (DAF) greater than 0.80 within 100 kb sliding windows is plotted for each population. Solid dots represent values above the 95th percentile for each population, whereas open dots are values below the 95th percentile. [file 1471-2148-10-267-S8.PDF]

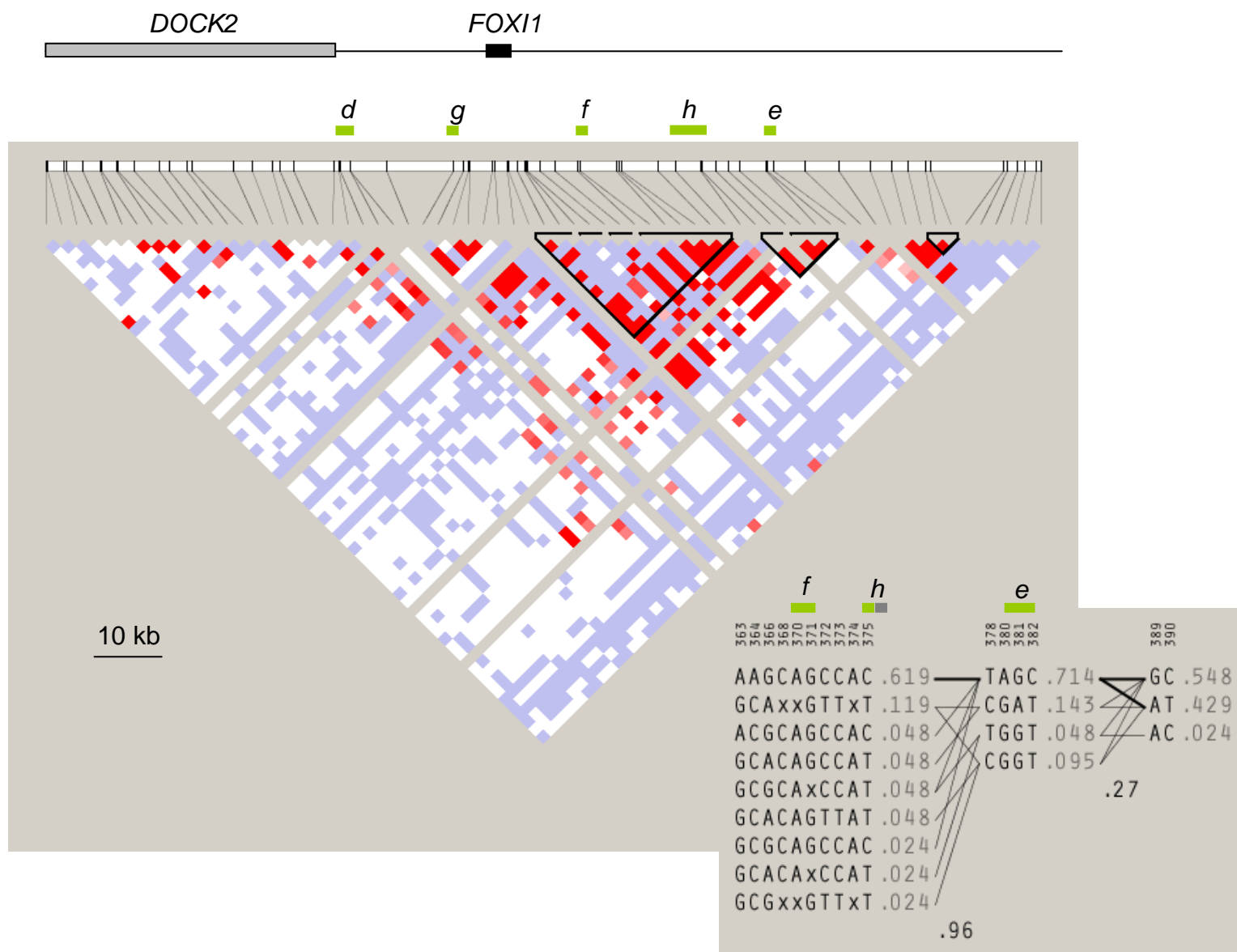

Supplement: Additional file 9 — Figure S6: Pattern of linkage disequilibrium around FOXI1 in the Yoruba population from the CEPH-HGDP diversity panel. Linkage disequilibrium from position 169,401,507 to 169,544,856 on chromosome 5, NCBI build 36.3. Green boxes represent significant core haplotypes from the LRH test and are labelled with letters d-h as in Table 4. [file 1471-2148-10-267-S9.PDF]
