# Supplementary material for: African signatures of recent positive selection in human FOXI1
Source: BMC Evol Biol. 2010 Sep 1;10:267. doi: 10.1186/1471-2148-10-267 (PMC2939579; doi:10.1186/1471-2148-10-267)
Supplement: Additional file 4 — Figure S2: Alignment of the five FOXI1 mammalian sequences used in PAML analysis. [file 1471-2148-10-267-S4.pdf]

Multiple species alignment of the FOXI1 gene coding sequence

Before deleting alignment gaps. 1137 sites

```
human  ATG AGC TCC TTC GAC CTG CCG GCG CCC TCC CCA CCT CGC TGC AGC CCC CAG TTC CCC AGC ATC GGC CAG GAG CCC CCC GAG ATG AAC CTC TAC TAT GAG AAC TTC TTC CAC CCA CAG GGC GTG CCC AGC CCT CAG CGG CCC --- TCC TTC
chimp  ATG AGC TCC TTC GAC CTG CCG GCG CCC TCC CCA CCT CGC TGC AGC CCC CAG TTC CCC AGC ATC GGC CAG GAG CCC CCC GAG ATG AAC CTC TAC TAT GAG AAC TTC TTC CAC CCA CAG GGC GTG CCC AGC CCT CAG CGG CCC --- TCC TTC
mouse  ATG AGC TCC TTC GAC CTC CCA GCG CCT TCA CCA CCT CGG TGC AGT CCC CAG TTC CCC AGC ATC GGC CAG GAG CCC CCG GAG ATG AAT CTT TAC TAT GAG AAT TTC TTC CAC CCT CAG GGC ATG CCC AGT CCT CAG CGC CCA ACC TCC TTC
rat    ATG AGC TCC TTC GAC CTC CCG GCG CCC TCG CCA CCT CGA TGC AGC CCC CAG TTC CCC AGC ATC GGC CAG GAG CCC CCC GAG ATG AAT CTT TAC TAT GAG AAC TTC TTC CAC CCT CAG GGC ATG CCC AGT CCT CAG CGC CCC ACC TCC TTC
dog    ATG AGC GCC TTC GAC CTC CCG GCG CCC TCC CCA CCT CGC TGC AGC CCC CAG TTC CCC AGC ATC GGC CAG GAG CCC CCC GAG ATG AGC CTT TAC TAT GAG AAC TTC TTC CAC CCC CAG GGC GTG CCC AGC CCT CAG CGG CCC CCC TCC CTT

human  GAG GGG GGC GGC GAG TAT GGG GCC ACC CCC AAC CCC TAC CTC TGG TTC AAC GGG CCC ACC ATG ACC CCG CCA CCC TAC CTG CCC GGC CCC AAC GCC AGC CCC TTC CTG CCC CAG GCC TAT GGA GTG CAG AGG CCG CTG CTG CCC AGC GTG
chimp  GAG GGG GGC GGC GAG TAT GGG GCC ACC CCC AAC CCC TAC CTC TGG CTC AAC GGG CCC ACC ATG ACC CCG CCA CCC TAC CTG CCC GGC CCC AAC GCC AGC CCC TTC CTG CCC CAG GCC TAT GGA GTG CAG AGG CCG CTG CTG CCC AGC GTG
mouse  GAA GGT GGT GGC GAA TAC GGG ACC ACT CCC AAC CCC TAC CTC TGG CTC AAT GGG CCG GCC ATG ACC CCA CCA CCC TAC CTG CCT GGC ACT AAC GCC AGC CCC TTT CTA CCC CAG GCC TAT GGC ATG CAG AGG CAG CTA CTG CCC --- ---
rat    GAA GGT GGT GGC GAG TAC GGG GCC ACC CCC AAC CCC TAC CTC TGG CTC AAT GGG ACA GCC ATG ACC CCA CCA CCC TAC CTG CCC GGC ACT AAC GCC AGC CCC TTC TTA CCC CAG GCC TAC GGC ATG CAG AGG CAG CTA TTG CCC --- ---
dog    GAG GGG GGC GGC GAG TAC GGG GCC CCC CCC AAC CCC TAC CTG TGG CTC AAC GGG CCT GCC ATG ACT CCG CCA CCC TAC CTG CCC GGC GCC AAC GCC AGC CCC TTC CTG CCC CAG GCC TAC GGT GTG CAG AGG CAG CTG CTG CCC GGC GTG

human  TCG GGG CTT GGG GGG AGC GAC CTG GGC TGG CTG CCC ATC CCC TCG CAG GAG GAG CTG ATG AAG CTG GTG CGG CCA CCC TAT TCC TAC TCG GCT CTC ATC GCC ATG GCC ATC CAC GGG GCA CCC GAC AAG CGC CTC ACT CTC AGC CAG ATC
chimp  TCG GGG CTT GGG GGG AGC GAC CTG GGC TGG CTG CCC ATC CCC TCG CAG GAG GAG CTG ATG AAG CTG GTG CGG CCA CCC TAT TCC TAC TCG GCT CTC ATC GCC ATG GCC ATC CAC GGG GCA CCC GAC AAG CGC CTC ACT CTC AGC CAG ATC
mouse  --- --- --- --- --- AGC GAC CTG GGC TGG CTG CCC ATC CCC TCT CAG GAA GAG CTC ATG AAG CTG GTA CGC CCA CCA TAT TCC TAT TCA GCC CTC ATT GCC ATG GCA ATC CAC GGT GCG CCC GAC CAG CGC CTC ACA CTG AGC CAG ATC
rat    --- --- --- --- --- AGT GAG CTG GGC TGG CTG CCC ATC CCC TCT CAG GAG GAG CTC ATG AAG CTG GTG CGC CCA CCA TAT TCC TAC TCT GCG CTT ATT GCT ATG GCA ATC CAC GGT GCT CCT GAC CAG CGT CTC ACA CTG AGC CAG ATC
dog    TCT GGG CTG GGG GGC GGC GAC CTG GGC TGG CTG CCC ATC CCC TCG CAG GAG GAG CTG ATG AAG CTG GTG CGG CCC CCC TAT TCC TAC TCG GCT CTC ATT GCC ATG GCC ATC CAC GGG GCG CCC GAC AAG CGC CTC ACC CTC AGC CAG ATC

human  TAC CAG TAC GTG GCC GAC AAC TTC CCC TTC TAC AAC AAG AGC AAG GCC GGC TGG CAG AAC TCC ATC CGC CAC AAC CTG TCG CTC AAC GAC TGC TTC AAG AAG GTG CCC CGC GAC GAG GAC GAC CCG GGC AAA GGG AAT TAC TGG ACC CTG
chimp  TAC CAG TAC GTG GCG GAC AAC TTC CCC TTC TAC AAC AAG AGC AAG GCC GGC TGG CAG AAC TCC ATC CGC CAC AAC CTG TCG CTC AAC GAC TGC TTC AAG AAG GTG CCC CGC GAC GAG GAC GAC CCG GGC AAA GGG AAT TAC TGG ACC CTG
mouse  TAC CAG TAC GTG GCC GAC AAC TTT CCC TTT TAC AAC AAG AGC AAG GCT GGC TGG CAG AAC TCC ATC CGC CAC AAC CTG TCA CTC AAC GAC TGC TTC AAG AAG GTG CCC CGA GAT GAG GAC GAC CCA GGC AAA GGG AAT TAC TGG ACT CTG
rat    TAC CAG TAC GTG GCC GAC AAC TTC CCC TTT TAC AAC AAG AGC AAA GCT GGC TGG CAG AAC TCC ATC CGC CAC AAC CTG TCA CTC AAC GAC TGC TTC AAG AAG GTG CCC CGA GAT GAG GAC GAC CCA GGC AAA GGG AAT TAC TGG ACT CTG
dog    TAC CAG TAC GTG GCC GAC AAC TTC CCC TTC TAC AAT AAG AGC AAG GCC GGC TGG CAA AAC TCC ATC CGC CAC AAC CTG TCT CTC AAT GAC TGC TTC AAG AAG GTG CCC CGC GAT GAG GAC GAC CCG GGC AAA GGG AAT TAC TGG ACC TTG

human  GAC CCC AAC TGT GAG AAA ATG TTC GAC AAT GGA AAT TTC CGC AGG AAA AGG AAG AGA AAA TCA GAT GTT TCC TCT AGC ACA GCC TCC TTG GCC TTA GAG AAG ACA GAG AGC AGT CTC CCG GTG GAC AGC CCC AAG ACC ACG GAG CCT CAG
chimp  GAC CCC AAC TGT GAG AAA ATG TTC GAC AAT GGA AAT TTC CGC AGG AAA AGG AAG AGA AAA TCA GAT GTT TCC TCC AGC ACA GGC TCC TTG GCC TTA GAG AAG ACA GAG AGC AGT CTC CTG GTG GAC AGC CCC AAG ACC ACG GAG CCT CAG
mouse  GAC CCT AAC TGT GAG AAG ATG TTT GAC AAC GGA AAC TTT CGC AGG AAG AGA AAG AGA AAA TCA GAT TCA TCC TCC AGC ACC AGC TCA TTG GCT TCA GAG AAA ACG GAG AAC GGT CTC CTG GCC AGC AGC CCC AAG CCC ACA GAG CCT CAG
rat    GAC CCC AAC TGT GAG AAG ATG TTT GAC AAC GGA AAC TTC CGC CGG AAG AGG AAG AGA AAA TCA GAT GCA TCC TCC AGC ACC GGC TCC TTG GCT TCT GAG AAA ACG GAG AAC AGG CTC CTG TCC AGC AGC CCC AAG CCC ACA GAG CCT CAG
dog    GAC CCC AAC TGT GAG AAG ATG TTC GAT AAT GGA AAT TTC CGC AGG AAG AGG AAG AGG AAA TCG GAT GTT TCC TCC AGC GCG GGA TCC CTG GCC TCC GAG AAG ACA GAG AGC AGC CTC CTG GCG GGC AGC CCC AGG ACG ACG GAG GCC CAG

human  GAC ATC TTG GAT GGA GCC TCA CCA GGG GGC ACC ACC AGC TCC CCA GAG AAG CGG CCC TCC CCT CCC CCA TCA GGC GCC CCT TGC CTT AAC AGC TTC CTT TCC TCT ATG ACA GCC TAT GTG AGC GGG GGG AGC CCC ACG AGC CAC CCC TTG
chimp  GAC ATC TTG GAT GGA GCC TCA CCA GGG GGC ACC ACC AGC TCC CCA GAG AAG CGG CCC TCC CCT CCC CCA TCA GGC ACC CCT TGC CTT AAC AGC TTC CTT TCC TCT ATG ACA GCC TAT GTG AGC GGG GGG AGC CCC ACG AGC CGC CCC TTG
mouse  GAG GTC TTA GAC ACC GCC TCG CCA GAC ACT ACC ACC AGC TCC CCA GAG AAG CGG TCC TCC CCT GCC CCC TCG GGC ACC CCG TGT CTC AAC AAC TTC CTC TCC ACC ATG ACA GCA TAT GTG AGT GGG ACA AAC CCT ATT AGC CGC TCC GTG
rat    GAG GTC TTA GAC ACT GCC TCA CCA GAC ACC ACC AGC AGC TCC CCA GAG AAG CGG TCC TCC CCT GCT CCC TCA GGC ACC CCG TGT CTC AAC AAC TTC CTC TCT ACC ATG ACA GCC TAT GTG AAT GGG ACA AAC CCC ATT AGC CGC TCA GCA
dog    GAC ATC TTG GAC AGT GCC TCC CCA GGG --- ACC GCC AGC CCC CCC GAG AAG CAG TCC TCG CCT CCC CCA CCC GGC ACC CCA TGC CTC AAC AGC TTC CTC TCC TCC ATG ACC TCC TAT GTG AGC GGG TCG AGC CCA GTG AGC CGC CCT GTG

human  GTC ACA CCA GGA CTG AGC CCT GAG CCC AGT GAC AAG ACG GGG CAG AAC TCA CTG ACC TTC AAC TCC TTC TCC CCG CTC ACC AAC CTC AGC AAC CAC AGC GGT GGG GGT GAC TGG GCG AAC CCC ATG CCC ACC AAC ATG CTC AGC TAT GGA
chimp  GTC ACA CCA GGA CTG AGC CCT GAG CCC AGT GAC AAG ACA GGG CAG AAC TCA CTG AGC TTC AAC TCC TTC TCC CCG CTC ACC AAC CTC AGC AAC CAC AGC GGT GGG GGT GAC TGG GCG AAC CCC ATG CCC ACC AAC ACG CTC AGC TAC GGA
mouse  GCC ACA CCA GGA CTG AGC TCA GAG CCT ATT GAC AAG ATG GGG CAG AAC TCA CTG AAT TTC AAC TCC TAC ACC CCC CTT ACC AAC CTC AGT AGC CAT GGG AAT GGG GGT GAA TGG GCC AAC CCA GTA GCC ACC AAT GCT CTG GGC TAT GGA
rat    GCC ACA CCA GGA CTG AGC CCG GAG CCT GTT GAC AAG ATG GGG CAG AAC TCA CTG AAT TTC AAC TCC TAC ACC CCC CTT ACC AAC CTC AGT AGC CAT GGG AAT GGG GGT GAA TGG GCC AAC CCG GTA CCC ACC AAT GCT CTG GGC TAT GGA
dog    GCA ACA CCA GGA CTG AGC CCT GAG CCC ACT GAC AAA ACG GGG CAG AAC TTG CTG AAC TTC AGC TCC TAC ACC CCA CTC ACC AAC CTC AGC AGC CAT GGG GGT GGG GGC GAA TGG GCC AAC CCC ATG CCC GCC AAC ACT CTC AGC TAC GGG

human  GGA TCT GTG CTC AGC CAA TTC AGC CCT CAC TTC TAC AAC AGT GTC AAC ACC AGT GGT GTC CTC TAC CCC AGG GAG GGC ACC GAG GTC
chimp  GGA TCT GTG CTC AGC CAA TTC AGC CCT CAC TTC TAC AAC AGT GTC AAC ACC AGT GGT GTC CTC TAC CCC AGG GAG GGC ACC GAG GTC
mouse  GGC TCC GTC TTC AAC CAG TTC AGT CCA CAT TTC TAC AAC AGC ATT AAC ACT AAC GGT ATC CTA TTC CCC AGG GAA GGC ACT GAA GTC
rat    GGC CCT GTC TTC AAC CAG TTC AGT CCA CAT TTC TAC AAT AGC ATC AAC ACC AAC AGT ATC CTC TTC CCC AGG GAA GGC ACT GAA GTC
dog    GGC TCT GTC CTC AAC CAG TTC AGC CCT CAT TTC TAC AAC AGC ATC AAC ACA AAC AGT GTC CTC TAC CCC AGG GAG GGC ACT GAT GTC
```
