# Supplementary material for: African signatures of recent positive selection in human FOXI1
Source: BMC Evol Biol. 2010 Sep 1;10:267. doi: 10.1186/1471-2148-10-267 (PMC2939579; doi:10.1186/1471-2148-10-267)
Supplement: Additional file 5 — Table S3: Significance of the likelihood ratio tests of positive selection performed on the human lineage for the FOXI1 gene. [file 1471-2148-10-267-S5.PDF]

Table S3. Significance of the likelihood ratio tests of positive selection performed on the human lineage for the *FOXI1* gene.

Compared organisms	Branch test	Strict branch + site test
Hsa, Ppa, Ptr, Ggo, Ppy, Hkl, Mmu, Pha, Sla, Rno and Mms	0.18714	0.80650
Hsa, Ppa, Ptr, Mmu, Pha, Sla, Rno and Mms	0.59670	0.62421
Hsa, Ppa, Ptr, Ggo, Ppy, Hkl, Mmu, Pha, Rno and Mms	0.18714	0.08065
Hsa, Ppa, Ptr, Ggo, Ppy, Hkl, Mmu, Pha, Sla	0.15939	1.00000
Hsa, Ppa, Ptr, Ggo, Ppy, Hkl, Sla, Rno, Mms	0.18463	0.8065
Hsa, Ptr, Ppy, Mmu, Rno, Mms, Ocu, Cfa, Bta, Dno, Laf, Mdo	0.59670	0.84148

In all cases p = Chi dist (2X(lnL1-lnL2), (np1-np2)). The three letter species codes are (% coverage): Hsa (human, 100%), Ppa (bonobo, 81.31%), Ptr (chimp, 100%), Ggo (gorilla, 78.57%), Ppy (orangutan, 100%), Hkl (gibbon, 79.63%), Mmu (macaque, 100%), Pha (baboon, 15.34%), Sla (Tamarin, 75.13%), Rno (rat, 100%), Mms (mouse, 100%), Ocu (rabbit, 100%), Cfa (dog, 100%), Bta (cow, 100%), Dno (armadillo, 100%), Laf (elephant, 100%) and Mdo (opossum, 100%)
