# Supplementary material for: African signatures of recent positive selection in human FOXI1
Source: BMC Evol Biol. 2010 Sep 1;10:267. doi: 10.1186/1471-2148-10-267 (PMC2939579; doi:10.1186/1471-2148-10-267)
Supplement: Additional file 10 — Table S4: Functional characterization and allele frequencies for functionally relevant SNPs within ~ 140 kb containing the FOXI1 gene. [file 1471-2148-10-267-S10.PDF]

Table S4. Functional characterization and allele frequencies for functionally relevant SNPs within ~ 140 kb containing the *FOXI1* gene.

SNP ID	Position[a]	Alleles[b]	Africans	Europeans	Asians	Sources	Functional Effect[d]
rs6555882	169401666	G/**C**	0.683	1	1	HapMap-YRI, CEU, CHB, JPT	ESE (*DOCK2*)
rs17647491	169416284	**C**/T	1	0.966	1	HapMap-YRI, CEU, CHB, JPT	ESE (*DOCK2*)
rs13179480	169428747	**A**/C	1	1	0.988	HapMap-YRI, CEU, JPT	CNS, ESE (*DOCK2*)
rs13179490	169428777	**A**/C	1	1	1	HapMap-YRI, CEU, CHB, JPT	CNS (*DOCK2*)
rs2270900	169435654	**T**/C	1	1	0.989	HapMap-YRI, CEU, CHB, JPT	ESE (*DOCK2*)
rs1045168	169437321	**T**/C	0.619	0.758	0.895	HGDP-Yoruba, HapMap- CEU, JPT	ESE (*DOCK2*)
rs2270898	169441471	**T**/A	1	1	0.989	HapMap-YRI, CEU, CHB, JPT	CNS, ESE (*DOCK2*)
rs1045176	169442598	G/**T**	0.595	0.121	0.478	HapMap-YRI, CEU, CHB	ESE (*DOCK2*)
rs9307	169442601	A/**G**	0.080	0.361	0.330	1000 genomes (dbSNP)-YRI, CEU, CHB, JPT	ESE (*DOCK2*)
rs17072089	169461251	**C**/G	0.978	0.979	1	Afr-Am, European, Asian (Perlegen)	New TFBS (*FOXI1*)
rs7704953	169461667	C/**T**	0.929	0.583	0.818	HGDP-Yor, HapMap- JPT, 1000 ge (dbSNP)-CEU	New TFBS (*FOXI1*)
rs34218925	169465509	G/T	NA[c]	NA[c]	NA[c]		ESE (*FOXI1*)
rs2277944	169465818	A/**G**	0.714	0.188	0.479	HGDP-Yor, European, Asian (Perlegen)	ESE (*FOXI1*)
rs35678180	169467782	G/A	0.974	NA[c]	NA[c]	Afr-Am (Applera)	ESE (*FOXI1*)
rs10063424	169468100	T/**C**	0.024	0.092	0.044	HGDP-Yoruba, HapMap- CEU, CHB	ESE (*FOXI1*)
rs3828625	169468141	**T**/C	1	1	0.988	HGDP-Yoruba, HapMap- CEU, JPT	CNS, ESE (*FOXI1*)
rs6873124	169468312	A/**C**	0.575	0.164	0.489	HapMap-YRI, CEU, CHB	ESE (*FOXI1*)
rs6555887	169468633	**A**/G	0.847	0.908	0.978	HapMap-YRI, CEU, JPT	ESS, ESE, miRNA (*FOXI1*)
rs6555888	169468728	G/**A**	0.842	0.808	0.944	HapMap-YRI, CEU, JPT	miRNA (*FOXI1*)

[a] SNP positions are based on NCBI build 36.3, [b]Ancestral allele in bold, [c]Not available, [d] Functional effect as predicted by PupaSuite [40, 41]: ESE, exonic splicing enhancer; CNS, coding non-synonymous SNPs; TFBS, transcription factor binding sites; ESS, exonic splicing silencer; miRNA, microRNAs and their targets.
